# MiR-30c-5p/ROCK2 axis regulates cell proliferation, apoptosis and EMT via the PI3K/AKT signaling pathway in HG-induced HK-2 cells

**DOI:** 10.1515/biol-2020-0089

**Published:** 2020-12-23

**Authors:** Lianshun Cui, Meiyan Yu, Xinglei Cui

**Affiliations:** Department of Kidney Disease of Internal, Weihai Central Hospital, No. 3, Mishandong Road West, Wendeng District, 264400, Weihai, China

**Keywords:** miR-30c-5p, ROCK2, diabetic nephropathy, proliferation, apoptosis, EMT

## Abstract

Diabetic nephropathy (DN) is one of the most common complications of diabetes mellitus. Increasing evidence suggests that microRNA-30c-5p (miR-30c-5p) participates in the pathogenesis of DN, but the mechanism has not been clearly understood. Therefore, this study aimed to investigate the biological role of miR-30c-5p in human DN progression *in vitro*. Compared with the controls, DN tissues and high glucose-induced HK-2 cells had significantly reduced miR-30c-5p levels, while ROCK2 expression was prominently elevated. Additionally, the miR-30c-5p mimic distinctly facilitated cell proliferation and blocked cell apoptosis and epithelial–mesenchymal transition (EMT). However, ROCK2 was a target gene of miR-30c-5p, and the effects of miR-30c-5p mimic on cell proliferation, apoptosis and EMT were reversed by ROCK2 upregulation *in vitro*. Furthermore, the pathogenesis of DN was regulated by the miR-30c-5p/ROCK2 axis via the PI3K/AKT pathway. MiR-30c-5p regulating cell proliferation, apoptosis and EMT through targeting ROCK2 via the PI3K/AKT pathway provides the novel potential target for clinical treatment of DN.

## 1 Introduction

Diabetic nephropathy (DN) is a major complication of diabetes mellitus, as well as the predominant cause of advanced renal disease [1]. Around 10–40% of patients with type 2 diabetes eventually develop DN in urban China [2]. However, the pathogenesis of DN is complex, influenced by multiple factors such as nonenzymatic saccharification, renal hemodynamic changes, hypertension, dyslipidemia, oxidative stress, protein kinase C activation, vasoactive substances and cytokines, as well as other genetic factors [3]. It seems that different types of kidney cells are sensitive to hyperglycemia in varying degrees [4]. Research on the pathogenesis of DN is particularly important to better understand the mechanism and thus develop more effective therapies.

MicroRNAs (miRNAs) have been shown to be a type of non-coding RNA with a length of 20–24 nucleotides (nts) [5]. MiRNAs are involved in a number of processes in the development of various diseases such as heart failure and cancer as well as diabetes [6,7,8]. Studies have reported that miRNAs are associated with some renal diseases. For example, miR-29c is a signature miRNA in high glucose (HG)-induced conditions, targeting sprouty homolog 1, and reduced expression of miR-29c prevents DN progression [9]. MiR-193a appears to induce focal segmental glomerulosclerosis by inhibiting the expression of Wilms’ tumor protein [10]. MiR-192 exerts its role in diabetic kidney glomeruli via inhibition of E-box repressors [11]. Moreover, miR-29s and miR-let-7s have been confirmed to function as key antifibrotic players in DN [12]. MiR-30c-5p in serum acts as a potential biomarker in multiple system atrophy [13]. Furthermore, miR-30c-5p has been revealed to be involved in reducing renal ischemia-reperfusion via macrophages [14]. In this study, the biological role of miR-30c-5p in DN was investigated using HG-induced HK-2 cells *in vitro*, which uncovered the possible mechanisms of miR-30c-5p’s effects on the progression of human DN.

Rho-associated coiled coil-containing protein kinase 2 (ROCK2) belongs to the Rho-associated kinase (ROCK) family. The ROCK family includes ROCK1 and ROCK2 members, and the high similarity of their amino acid sequences suggests that these two members perform many of the same functions [15]. Particularly, ROCK2 downregulation blocks HG-induced hyperpermeability in kidney glomerular endothelium [16]. Previous research indicates that ROCK2 is a critical regulator of axonal degeneration and neuronal death, as well as axonal regeneration in the central nervous system [17]. Thus, ROCK2 could be closely related to the development of multiple types of cancer, as well as diseases of the central nervous system and kidney dysfunction.

The underlying mechanism of ROCK2 in the progression of kidney diseases needs further exploration. Since phosphatidylinositol 3-kinase (PI3K) and protein kinase B (AKT) play vital roles in several cellular processes, including proliferation, apoptosis, migration and glucose metabolism [18,19], we hypothesized that the PI3K/AKT signaling pathway participates in the progression of DN.

Herein, we measured the level of miR-30c-5p and ROCK2 expression in DN tissues and in an HG-induced DN cell model. The regulatory mechanism of miR-30c-5p and ROCK2 in the pathogenesis of DN *in vitro* was investigated.

## 2 Materials and methods

### 2.1 Kidney tissue samples

Human DN tissue samples (*n* = 40) and the adjacent normal kidney tissues (*n* = 40) were donated by DN patients at Weihai Central Hospital. The DN patients included 18 males and 22 females, ranging from 40 to 65 years. All the patients were diagnosed and classified according to the World Health Organization diagnostic criteria for diabetes [20]. The DN patients underwent a negative urine protein test, with 30–300 mg/24 h urine albumin, indicating early renal damage. Moreover, the DN patients were diagnosed with the presence of specific nodular glomerulosclerosis of diabetes mellitus (the Kimmelstiel–Wilson lesion). We did not find other primary and secondary factors or complications causing renal damage.


**Informed consent:** Informed consent has been obtained from all individuals included in this study.
**Ethical approval:** The research related to human use has been complied with all the relevant national regulations, institutional policies and in accordance with the tenets of the Helsinki Declaration and has been approved by the Ethics Committee of Weihai Central Hospital.

### 2.2 Cell culture and treatment

Human kidney cells HK-2 were purchased from the Chinese Academy of Sciences Shanghai Cell Bank (Shanghai, China). HK-2 cells were cultured in Dulbecco’s modified Eagle’s medium (DMEM; Thermo Fisher Scientific, Rockford, IL, USA) supplemented with 5.6 mmol/L glucose (Sigma, St. Louis, MO, USA), 10% fetal bovine serum (Gibco, Carlsbad, CA, USA), 100 U/mL penicillin and 100 µg/mL streptomycin, in an incubator with 5% CO_2_ at 37°C.

To establish the DN cell model, HK-2 cells were trypsinized, and 2 mL of cell suspension was added to each well of a six-well culture plate at a density of 1 × 10^6^ cells/mL. Then, the cells were incubated in serum-free DMEM for 12 h until 70–80% confluence. HK-2 cells were treated with normal glucose (NG; final concentration of d-glucose 5.6 mmol/L), HG (final concentration of d-glucose 30 mmol/L) and high osmotic pressure control group (HO; 5.6 mmol/L d-glucose + 24.4 mmol/L d-mannitol).

### 2.3 Transient transfection

HK-2 cells were seeded in a six-well plate. When cells grew to 60–85% confluence, they were transfected with vectors or oligonucleotides using Lipofectamine™ 2000 (Invitrogen, Carlsbad, CA, USA) following the manufacturer’s instructions. Then, the HK-2 cells were cultured with NG, HG or HO after transfection for 6 h. The HK-2 cells in the mock group were treated with no sequences, and the HK-2 cells in the NC group were transfected with control miR-30c-5p. The vectors and oligonucleotides used were as follows: overexpression vector of ROCK2 (pcDNA-ROCK2), pcDNA 3.1 empty vector (pcDNA-control), small interfering RNA against ROCK2 (si-ROCK2), miR-30c-5p mimic (5′-UGUAAACAUCCUACACUCUCAGC-3′), miR-30c-5p inhibitor (5′-GCUGAGAGUGUAGGAUGUUUACU-3′) and miR-30c-5p negative control (miR-control, 5′-CUAACGCAUGCACAGUCGUACG-3′). The above sequences were synthesized by GenePharma (Shanghai, China).

### 2.4 Quantitative reverse transcription-polymerase chain reaction (qRT-PCR)

Total RNA was extracted from tissues and cells using TRIzol (Invitrogen) in accordance with the manufacturer s instructions. Subsequently, reverse transcription was conducted using an ALL-in-one miRNA reverse transcription kit (GeneCopoeia, Rockville, MD, USA) according to the manufacturer’s instructions. The obtained cDNA was temporarily maintained at −80°C or directly used. qPCR was carried out using a SYBR® Premix Ex TaqTM II Kit (TaKaRa, Dalian, China) and ABI 7500 PCR instrument (Applied Biosystems, Rockford, IL, USA). Finally, mRNA expression ratio was calculated using the 2^−ΔΔCt^ method, and U6 and glyceraldehyde 3-phosphate dehydrogenase (GAPDH) were used as the internal reference. The following primers were used: miR-30c-5p (forward: 5′-GCCGCTGTAAACATCCTACACT-3′ and reverse: 5′-GTGCAGGGTCCGAGGT-3′), ROCK2 (forward: 5′-GCCGCCGTTGCCATATTAAG-3′ and reverse: 5′-CTGGAGCTGGGGGCTTTTTA-3′), GAPDH (forward: 5′-GGTCACCAGGGCTGCTTTTA-3′ and reverse: 5′-TTCCCGTTCTCAGCCTTGAC-3′), and U6 (forward: 5′-CTCGCTTCGGCAGCACA-3′ and reverse: 5′-AACGCTTCACGAATTTGCGT-3′).

### 2.5 Western blot assay

HK-2 cells and tissues were harvested and lysed in RIPA lysis buffer (Millipore, Bedford, MA, USA) containing protease and phosphatase inhibitors. Then, total protein concentration was evaluated with a BCA Protein Assay Kit (Sangon Biotech, Shanghai, China), and loading buffer was used for modulating the volume of protein solution. Briefly, 20 µL of solution containing 30 µg of protein was added to each well of a 10% sodium dodecyl sulfate-polyacrylamide gel. The separated protein was electro-transferred onto polyvinylidene difluoride membranes (Millipore), which were then blocked with 5% bovine serum albumin (Sangon Biotech). Then, the membranes were incubated with primary antibodies: ROCK2 (1:1,000, ab71598; Abcam, Cambridge, MA, USA), E-cadherin (1:1,000, ab76055; Abcam), vimentin (1:1,000, ab8979; Abcam), α-smooth muscle actin (α-SMA; 1:1,000, ab108424; Abcam), snail1 (1:1,000, ab53519; Abcam), transforming growth factor β 1 (TGFB1; 1:1,000, ab92486; Abcam), PI3K (1:1,000, ab151549; Abcam), phosphorylated PI3K (p-PI3K; 1:1,000, ab182651; Abcam), AKT (1:1,000, ab64148; Abcam), phosphorylated AKT (p-AKT; 1:500, ab8933; Abcam) and GAPDH (1:1,000, ab8245; Abcam) overnight at 4°C. After washing, the membranes were incubated with a secondary antibody (1:5,000, ab205718 or ab205719; Abcam) for 1 h at room temperature. Subsequently, a Chemiluminescence Reagent Kit (Millipore) and a ChemiDoc MP Imaging System (Bio-Rad, Philadelphia, PA, USA) were used to visualize protein bands.

### 2.6 3-(4,5-Dimethylthiazol-2-yl)-2,5-diphenyltetrazolium bromide (MTT) assay

HK-2 cells were counted, and 5 × 10^3^ cells were plated in a 96-well plate. After the cells were cultured for less than 24 h, they were transfected with vectors or oligonucleotides under the conditions of NG, HG or HO. Then, the cells were incubated for 24, 48, 72 and 96 h; the supernatant was discarded. MTT solution (20 µL) was added to a final concentration of 5 mg/mL and the cells were incubated for another 4 h at 37°C. Subsequently, 200 µL of DMSO was added into each well; formazan crystals were fully dissolved by oscillating the plate for 10 min. Finally, the optical density (OD) value in each well was measured at a wavelength of 490 nm using a Microplate Reader (Bio-Rad), and the OD of the blank well was regarded as zero.

### 2.7 Flow cytometry for analyzing cell apoptosis

An Annexin V-FITC Apoptosis Detection Kit (Beyotime, Shanghai, China) was used to detect cell apoptosis. HK-2 cells treated under different treatment conditions and in different transfected groups were washed twice with phosphate buffered saline on ice (Gibco), and then diluted with a 1× binding buffer to a final concentration of approximately 1 × 10^6^ cells/mL. Subsequently, 5 µL of Annexin V-FITC and propidium iodide (PI) was added to the labeled cell tubes, and the cells were stained for 15 min at room temperature in the dark. Finally, cell apoptosis was detected using flow cytometer (BD Biosciences, San Jose, CA, USA).

### 2.8 Dual-luciferase reporter assay

TargetScan predicted that ROCK2 might be a target of miR-30c-5p. Thus, a dual-luciferase reporter assay was conducted to confirm the direct interaction between miR-30c-5p and ROCK2. Common fragments of the synthetic wild-type (ROCK2 3′-UTR WT) or mutant (ROCK2 3′-UTR MUT) of ROCK2 3′-UTR were introduced into a pGL3-Basic vector (Promega, Madison, WI, USA). Subsequently, the correctly sequenced ROCK2 3′-UTR WT and ROCK2 3′-UTR MUT were co-transfected into HK-2 cells, and the cells were collected after transfection for 48 h. Then, luciferase assay reagent and Renilla assay buffer were dissolved with Dual-Luciferase Reporter Assay System Kit reagent (Promega). Ten microliters of cell lysate were added to each well of an opaque 96-well plate before the measurement of the luciferase and Renilla activities. Meanwhile, the firefly activities, as the indicator of the reporter gene, were identified by Luciferase Assay Reagent II, and the internal reference of Renilla activity was measured using Stop&Glo Reagent. The reporter gene was described via counting the ratio of firefly activity/Renilla activity.

### 2.9 Statistical analysis

The data were analyzed using SPSS 19.0 software and presented as mean ± standard deviation of three independent experiments. Student’s *t*-test and one-way analysis of variance were employed to examine the difference of pair groups and multiple groups, respectively. Statistical significance was considered at *P* < 0.05.

## 3 Results

### 3.1 Level of miR-30c-5p was effectively decreased, whereas ROCK2 expression was significantly increased in DN tissues

**Figure 1:**
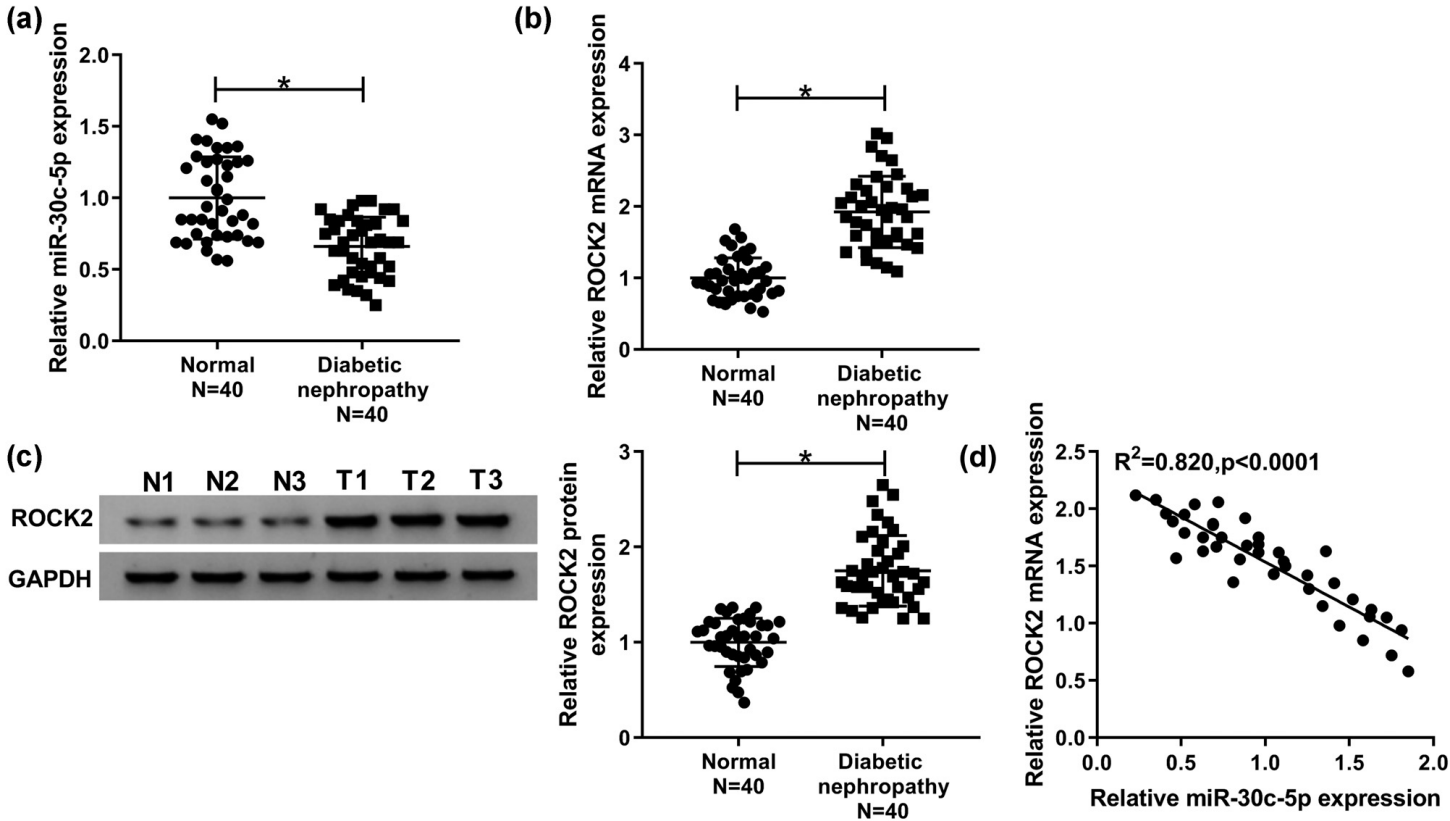
Level of miR-30c-5p was effectively decreased, whereas ROCK2 expression was significantly increased in DN tissues. (a and b) qRT-PCR was carried out to analyze the miR-30c-5p level and the mRNA level of ROCK2 in DN tissues compared with the paired control. (c) Protein expression of ROCK2 was measured by a western blot assay. (d) Spearman’s correlation analysis showed an inverse correlation between miR-30c-5p and ROCK2 mRNA levels. *P < 0.05.

In order to investigate the biological roles of miR-30c-5p and ROCK2 in DN development, the levels of miR-30c-5p and ROCK2 were analyzed by qRT-PCR. The results demonstrated that miR-30c-5p was greatly downregulated, while ROCK2 was notably upregulated in DN tissues (Figure 1a and b). Furthermore, the ROCK2 protein level was increased in the DN group (Figure 1c). Moreover, there was a negative linear correlation between miR-30c-5p level and ROCK2 expression (Figure 1d). In brief, the data suggested that miR-30c-5p and ROCK2 may play vital roles in the progression of DN.

### 3.2 HG conditions induced ROCK2 but suppressed miR-30c-5p expression in HK-2 cells

**Figure 2:**
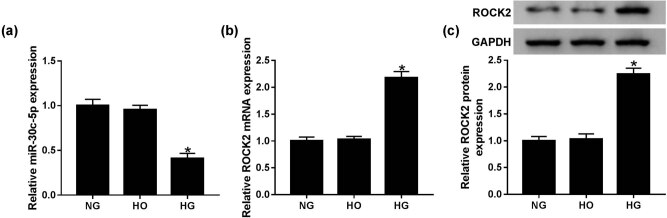
HG conditions in HK-2 cells induced ROCK2 expression but suppressed miR-30c-5p expression. (a and b) Levels of miR-30c-5p and ROCK2 were detected by qRT-PCR in HG-induced HK-2 cells. (c) Western blot assay was conducted to determine mature ROCK2 expression *in vitro*. NG: normal glucose (5.6 mmol/L d-glucose); HG: high glucose (30 mmol/L d-glucose); HO: high osmotic pressure solution (5.6 mmol/L d-glucose + 24.4 mmol/L d-mannitol). *P < 0.05.

To determine whether the expression of miR-30c-5p and ROCK2 in the HG-induced DN cell model was in line with the tissues, HK-2 cells were treated with NG, HG and HO. qRT-PCR results suggested that the level of miR-30c-5p was significantly reduced, while the ROCK2 mRNA level was increased in the HG group compared with that in NG and HO groups (Figure 2a and b). Western blot assay showed that the protein expression of mature ROCK2 was enhanced in the HG group (Figure 2c). These data show that the expression trend of miR-30c-5p and ROCK2 in HG-stimulated HK-2 cells is in line with that in DN tissues.

### 3.3 MiR-30c-5p promoted cell proliferation and repressed cell apoptosis and epithelial-mesenchymal transition (EMT) in HG-induced HK-2 cells

**Figure 3:**
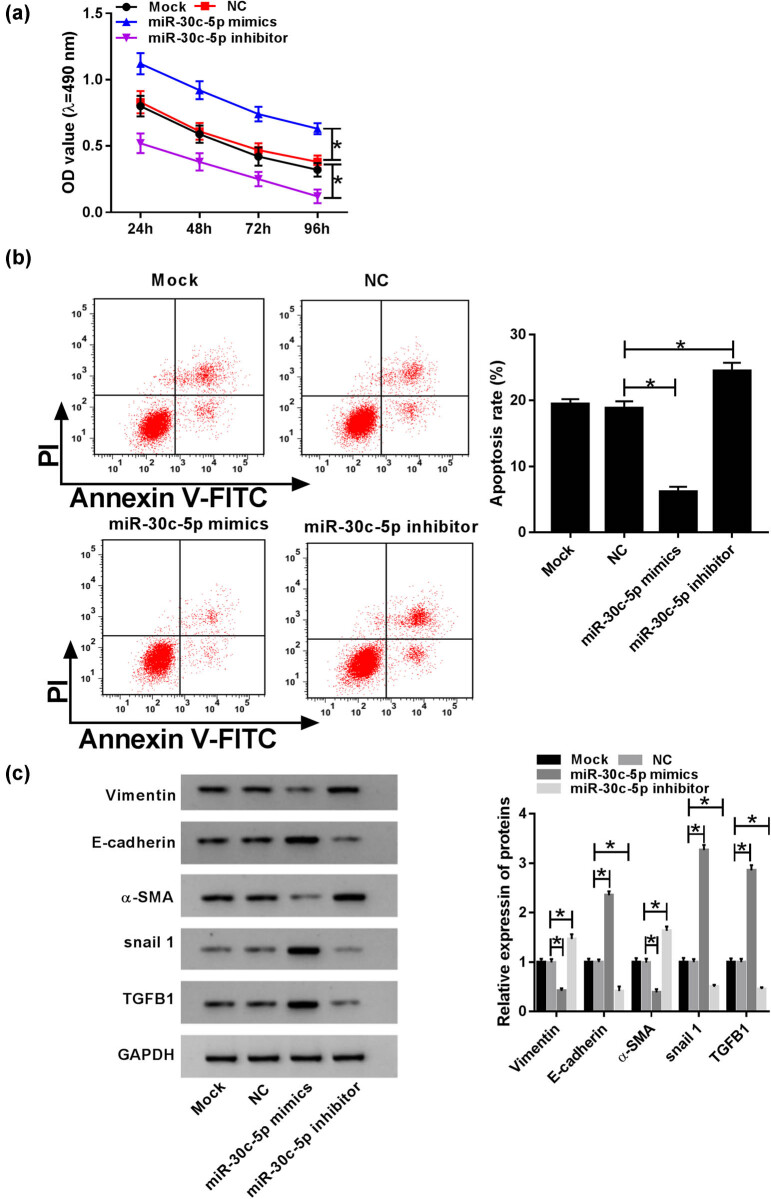
MiR-30c-5p promoted cell proliferation and repressed cell apoptosis and EMT in HG-induced HK-2 cells. (a) MTT assay was performed to evaluate cell proliferation in the HG-induced DN cell model. (b) Cell apoptosis was examined by flow cytometry. (c) Levels of EMT-related proteins vimentin, E-cadherin, snail1, TGFB1 and α-SMA were identified via a western blot assay. *P < 0.05.

Because of the low expression of miR-30c-5p in DN tissues, the assay aimed to investigate its potential biological role. An MTT assay was performed to detect cell proliferation of HK-2 cells under exposure to HG, and the result revealed that the proliferation of HK-2 cells in mock and NC groups was hindered with time, and there was no marked difference between these two groups. Simultaneously, the miR-30c-5p mimic dramatically promoted cell proliferation, whereas the miR-30c-5p inhibitor conspicuously restrained cell proliferation in HG-induced HK-2 cells (Figure 3a). As shown in Figure 3b, there was a distinct repression of cell apoptosis in HK-2 cells with the miR-30c-5p mimic, while the miR-30c-5p inhibitor prominently expedited the apoptosis rate in HK-2 cells. Furthermore, the boosted expression of E-cadherin, snail1 and TGFB1, as well as the inhibited levels of vimentin and α-SMA indicated that EMT was efficiently constrained by the miR-30c-5p mimic, while there was an opposite result caused by the miR-30c-5p inhibitor (Figure 3c). All evidence indicates that the miR-30c-5p mimic strikingly accelerates cell proliferation, while obviously impeding apoptosis and EMT in HG-stimulated HK-2 cells, while the miR-30c-5p inhibitor exerts an inverse effect.

### 3.4 ROCK2 was a target gene of miR-30c-5p

**Figure 4:**
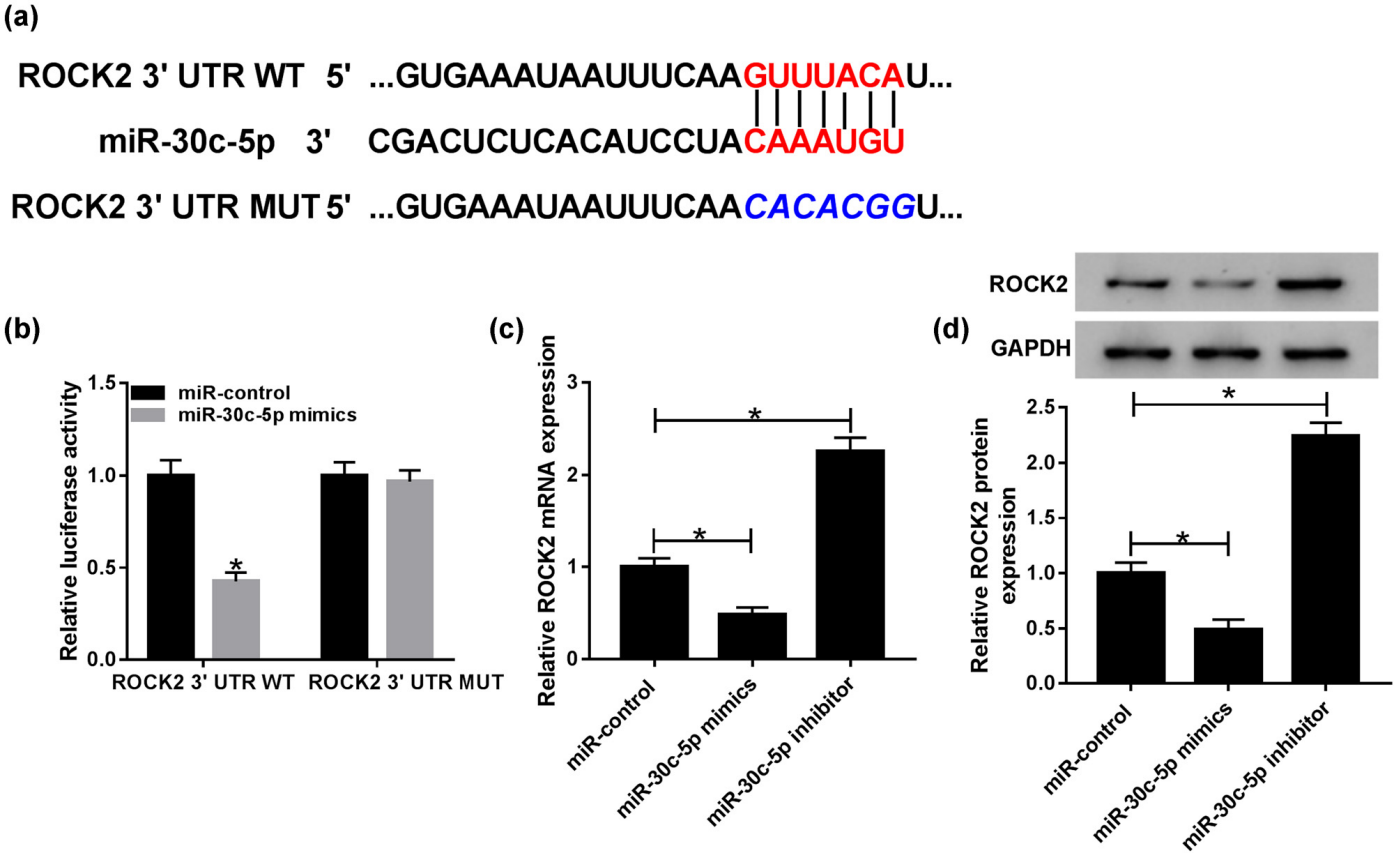
ROCK2 is a target gene of miR-30c-5p. (a) TargetScan was used to predict the interaction between miR-30c-5p and ROCK2. (b) The relationship between miR-30c-5p and ROCK2 was confirmed by a dual-luciferase reporter assay. (c and d) The expression of ROCK2 was measured post-transfection with the miR-control, miR-30c-5p mimic and miR-30c-5p inhibitor, respectively. *P < 0.05.

TargetScan predicted the short binding sites shared by miR-30c-5p and ROCK2 (Figure 4a). Subsequently, a dual-luciferase reporter assay was conducted to confirm the interaction between miR-30c-5p and ROCK2, and the results uncovered that luciferase activity is efficiently inhibited by ROCK2 3′-UTR WT, while no evident difference was seen in the ROCK2 3′-UTR MUT group (Figure 4b). Additionally, qRT-PCR and western blot assays were performed to investigate the regulatory mechanism between miR-30c-5p and ROCK2, and the expression of ROCK2 was markedly suppressed by miR-30c-mimic but apparently induced by the miR-30c-5p inhibitor (Figure 4c and d). In short, these data suggest that ROCK2 is a direct downstream gene of miR-30c-5p.

### 3.5 ROCK2 inhibited cell proliferation and enhanced cell apoptosis and EMT in HG-induced HK-2 cells

**Figure 5:**
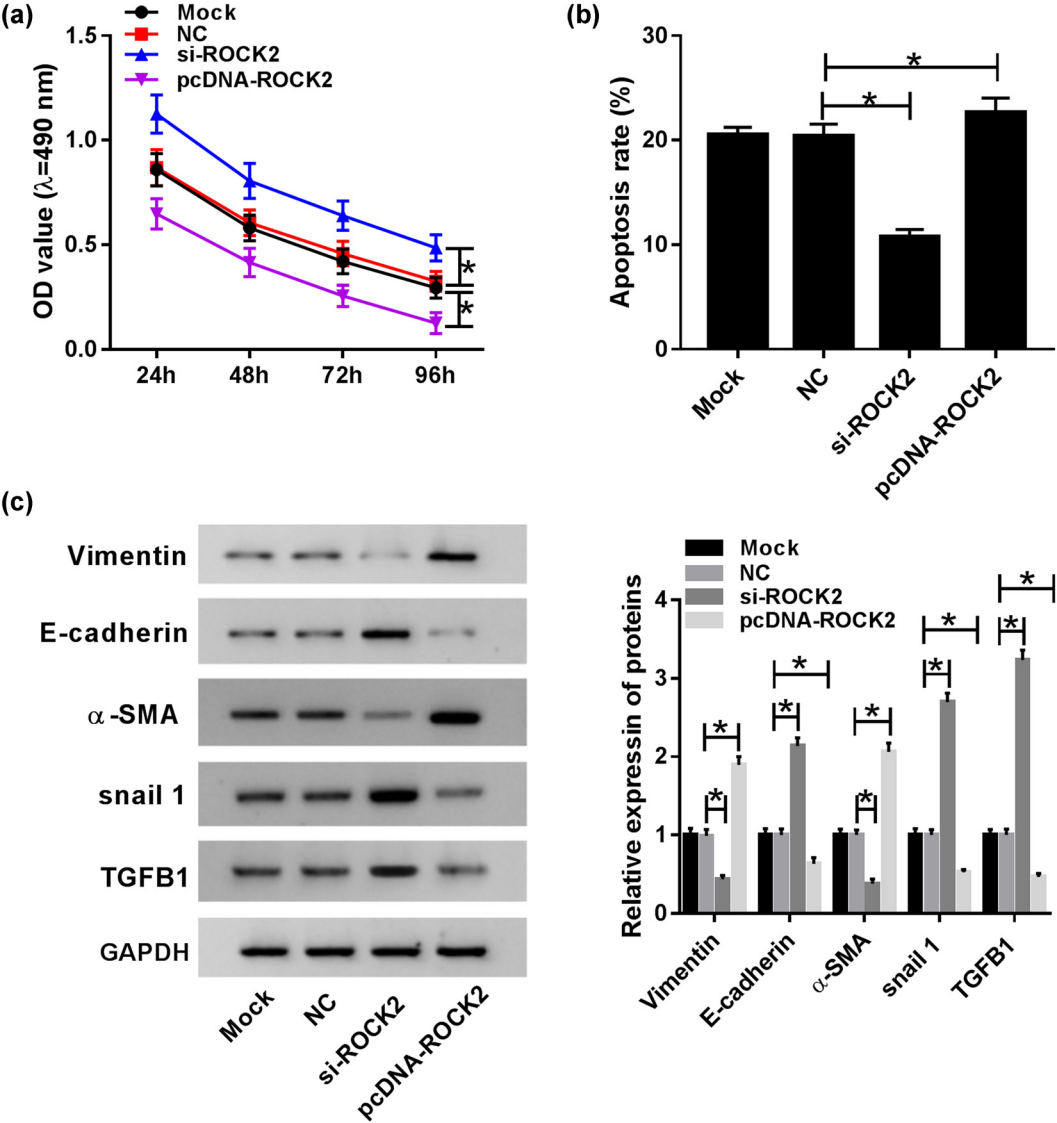
ROCK2 inhibited cell proliferation and enhanced cell apoptosis and EMT in HG-induced HK-2 cells. (a) Cell proliferation was analyzed by an MTT assay. (b) Flow cytometry was focused on HK-2 cell apoptosis. (c) The expression of EMT-related proteins including vimentin, E-cadherin, snail1, TGFB1 and α-SMA was assessed using a western blot assay. *P < 0.05.

To assess the therapeutic potential of ROCK2 in DN, si-ROCK2 and pcDNA-ROCK2 were transfected into HG-induced HK-2 cells. MTT assay results indicate that ROCK2 is an inhibitory factor in cell proliferation, and knockdown of ROCK2 evidently contributes to cell proliferation, as upregulation causes decreased cell proliferation in HG-induced HK-2 cells (Figure 5a). Moreover, cell apoptosis was dramatically repressed in the si-ROCK2 group, but apparently reinforced after transfection of pcDNA-ROCK2 in HG-treated HK-2 cells (Figure 5b). The EMT level was also determined through measuring the expression of vimentin, E-cadherin, α-SMA, snail1 and TGFB1, and the efficiently increased expression of E-cadherin, snail1 and TGFB1, and the conspicuously constrained expression of vimentin and α-SMA in the si-ROCK2 group suggest that knockdown of ROCK2 suppresses EMT in HG-induced HK-2 cells. The results of the pcDNA-ROCK2 group also support this conclusion (Figure 5c). These data reveal that knockdown of ROCK2 induces cell proliferation and suppresses apoptosis and EMT, whereas the function of ROCK2 overexpression in cell proliferation, apoptosis and EMT was opposite to that of ROCK2 silencing.

### 3.6 MiR-30c-5p exerted its function by targeting ROCK2 in the HG-stimulated DN cell model

**Figure 6:**
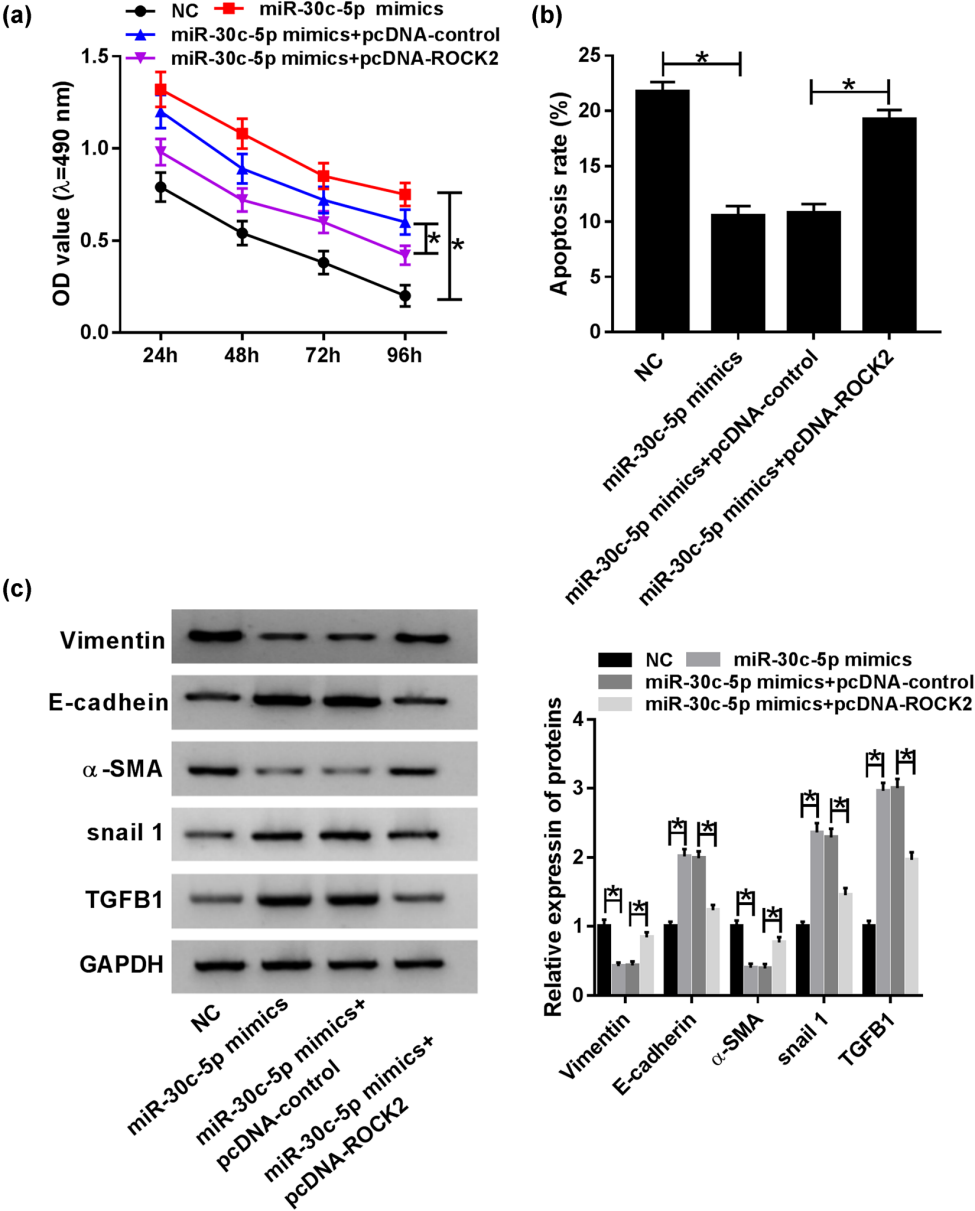
MiR-30c-5p exerted its function by targeting ROCK2 in the HG-stimulated DN cell model. (a) MTT assay was conducted to evaluate cell proliferation *in vitro*. (b) The impact of HG on cell apoptosis was analyzed via flow cytometry in HK-2 cells. (c) Western blot assay was used to determine mature protein expression of vimentin, E-cadherin, snail1, TGFB1 and α-SMA in HG-induced HK-2 cells. *P < 0.05.

Due to the inverse functions of miR-30c-5p and ROCK2, miR-30c-5p mimic, miR-30c-5p mimic + vector and miR-30c-5p mimic + pcDNA-ROCK2 were transfected into HG-stimulated HK-2 cells, respectively. An MTT assay discovered that the promotion effect of miR-30c-5p mimic on cell proliferation was abrogated by pcDNA-ROCK2 (Figure 6a). Synchronously, cell apoptosis suppressed by miR-30c-5p was reversed via pcDNA-ROCK2 *in vitro* (Figure 6b). In addition, the inhibitory effect of miR-30c-5p mimic on EMT was restrained by overexpression of ROCK2 in HG-induced HK-2 cells (Figure 6c). In brief, the effects of miR-30c-5p mimic on cell proliferation, apoptosis and EMT were relieved by upregulation of ROCK2 in the HG-induced DN cell model.

### 3.7 The effects of miR-30c-5p and ROCK2 were exerted on DN progression via the PI3K/AKT signaling pathway

**Figure 7:**
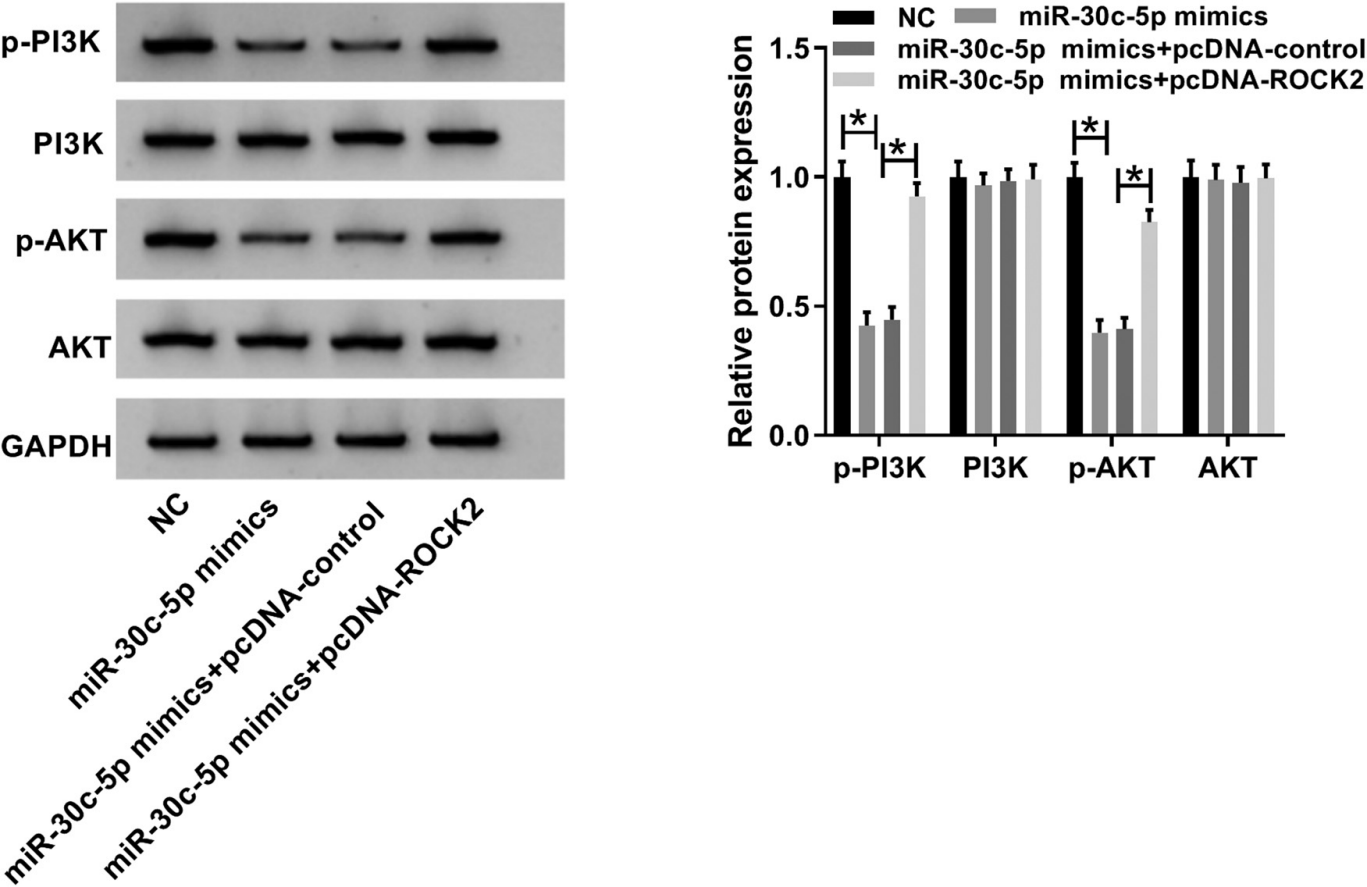
Effects of miR-30c-5p and ROCK2 were exerted on DN progression via the PI3K/AKT signaling pathway. The expression of the PI3K/AKT pathway-related proteins including p-PI3K, PI3K, p-AKT and AKT was detected using a western blot assay and quantified via Image J software. *P < 0.05.

In order to investigate the regulatory mechanism of miR-30c-5p and ROCK2 in the progression of DN, we detected the expression of p-PI3K, PI3K, p-AKT and AKT after transfection with miR-30c-5p mimic, miR-30c-5p mimic + vector and miR-30c-5p mimic + pcDNA-ROCK2 in HK-2 cells treated with HG. The results showed that the expressions of both p-PI3K and p-AKT were remarkably decreased in the miR-30c-5p mimic group, and they were improved after co-transfection with miR-30c-5p and pcDNA-ROCK2 *in vitro* (Figure 7). This indicates that the roles of miR-30c-5p and ROCK2 are carried out via the PI3K/AKT signaling pathway.

## 4 Discussion

It is well known that DN leads to end-stage kidney disease and increases all-cause mortality in diabetic patients [21]. According to the previous research, renal tubular damage is distinct in DN patients [22]. Here, HK-2 cells were treated with HG to cause damage, and the study found that the level of miR-30c-5p was obviously curbed in either HG-stimulated HK-2 cells or DN tissues. In contrast, ROCK2 expression was significantly augmented in the cell model and tissue samples. The aberrant expression of miR-30c-5p and ROCK2 showed their potential functions in the pathogenesis of human DN.

Emerging research indicates that miRNAs, such as miR-93 [23], miR-451 [24] and miR-23b [25], show aberrant expression in dysfunctional kidneys, including in DN. Understanding how miRNAs contribute to the progression of diseases is imminently important since several miRNAs are currently being explored as therapeutic agents for some diseases through early-stage clinical trials [26]. Moreover, miRNAs appear to be able to modify the expression of various target genes at transcription and post-transcription levels [27], and identification of classical or novel miRNA targets might provide unique therapeutic opportunities in different diseases. Based on the previous research examples, miR-27a is related to podocyte injury in DN via targeting Forkhead box protein O1 [28]. Over the past few decades, miR-30c has been proved to function as a tumor suppressor in colorectal carcinoma via regulating the target gene of B cell lymphoma 9 [29]. MiR-30c prevented diabetic cardiomyopathy by reducing the peroxisome proliferator-activated receptor alpha level [30]. Moreover, miR-30c-5p regulated neuropathic pain of rodents and was highly expressed in the spinal cord and dorsal root ganglia [31]. Furthermore, miR-30c-5p was associated with macrophage-mediated inflammation and pro-atherosclerosis signal pathways [32]. Increasing evidence reveals that EMT of glomerular endothelium is a modulation mechanism for potential cell dysfunction in endothelial injury [33]. In our study, miR-30c-5p was silent in DN tissue samples and HG-induced HK-2 cells. The miR-30c-5p mimic strikingly augmented cell proliferation, whereas it effectively hindered apoptosis and EMT in HG-induced DN cells. A previous study suggested that miR-29s and miR-let-7s exert antifibrotic effects on kidney fibrosis [12]. Consistently, miR-30c-5p has an antifibrotic effect through inhibiting EMT processes. This evidence suggested that miR-30c-5p participated in DN progression, and thus may be a novel target in clinical treatment of DN.

ROCK2 knockdown, which is regulated by miR-455-3p, suppressed renal fibrosis in DN [34], but it appears that the role of ROCK2 in the regulatory mechanism of diabetic heart disease is complex, referring to the phosphoinositide-dependent kinase-1/AKT signaling pathway [35]. Two members of the ROCK family have been associated with renal injury, e.g., a mouse model of unilateral ureteral obstruction nephropathy caused by tubulointerstitial fibrosis [36]. In this study, ROCK2 as the target factor was discovered to be directly targeted by miR-30c-5p. Furthermore, the promotion effect of the miR-30c-5p mimic on cell proliferation and inhibition effects on cell apoptosis and EMT were abolished by overexpression of ROCK2 in HG-stimulated HK-2 cells. Finally, the landmark proteins of the PI3K/AKT signaling pathway were also measured by a western blot assay, and the results demonstrated that miR-30c-5p and ROCK2 exerted roles at least partially via the PI3K/AKT signaling pathway in the progression of DN.

In summary, either the miR-30c-5p mimic or ROCK2 knockdown evidently induced cell proliferation, and notably restrained apoptosis and EMT in HG-stimulated HK-2 cells. Interestingly, the biological role of miR-30c-5p in cell proliferation, apoptosis and EMT was abrogated by overexpression of ROCK2 *in vitro*. Simultaneously, the miR-30c-5p/ROCK2 axis modulated the development of DN through the PI3K/AKT signaling pathway.

The level of miR-30c-5p was significantly curbed, while ROCK2 expression was effectively augmented in DN tissues. Moreover, HG-treated HK-2 cells were used to simulate a DN cell model in the study. Functionally, miR-30c-5p acted as a promoter of cell proliferation, while hindering cell apoptosis and EMT in HG-induced HK-2 cells. Additionally, the function of ROCK2 was opposite to that of miR-30c-5p in cell proliferation, apoptosis and EMT. Interestingly, ROCK2 was a target gene of miR-30c-5p and its overexpression abolished the effects of the miR-30c-5p mimic on cell proliferation, apoptosis and EMT in HG-stimulated HK-2 cells. Mechanically, the miR-30c-5p/ROCK2 axis regulated the progression of DN through the PI3K/AKT signaling pathway.
